# Enabling tradeoffs in privacy and utility in genomic data Beacons and summary statistics

**DOI:** 10.1101/gr.277674.123

**Published:** 2023-07

**Authors:** Rajagopal Venkatesaramani, Zhiyu Wan, Bradley A. Malin, Yevgeniy Vorobeychik

**Affiliations:** 1 Washington University in St. Louis, St. Louis, Missouri 63130, USA;; 2 Vanderbilt University Medical Center, Nashville, Tennessee 37212, USA

## Abstract

The collection and sharing of genomic data are becoming increasingly commonplace in research, clinical, and direct-to-consumer settings. The computational protocols typically adopted to protect individual privacy include sharing summary statistics, such as allele frequencies, or limiting query responses to the presence/absence of alleles of interest using web services called Beacons. However, even such limited releases are susceptible to likelihood ratio–based membership-inference attacks. Several approaches have been proposed to preserve privacy, which either suppress a subset of genomic variants or modify query responses for specific variants (e.g., adding noise, as in differential privacy). However, many of these approaches result in a significant utility loss, either suppressing many variants or adding a substantial amount of noise. In this paper, we introduce optimization-based approaches to explicitly trade off the utility of summary data or Beacon responses and privacy with respect to membership-inference attacks based on likelihood ratios, combining variant suppression and modification. We consider two attack models. In the first, an attacker applies a likelihood ratio test to make membership-inference claims. In the second model, an attacker uses a threshold that accounts for the effect of the data release on the separation in scores between individuals in the data set and those who are not. We further introduce highly scalable approaches for approximately solving the privacy–utility tradeoff problem when information is in the form of either summary statistics or presence/absence queries. Finally, we show that the proposed approaches outperform the state of the art in both utility and privacy through an extensive evaluation with public data sets.

The past several years have seen a sharp rise in the collection and sharing of genomic data as a result of advancements in personalized medicine technology in clinical settings, as well as the rising popularity of direct-to-consumer genetic testing. Data sharing in the former setting is usually controlled through a combination of technical safeguards in order to comply with privacy-protection laws, as well as data-sharing agreements (Wan et al. 2022). The latter contributes to sharing of genomic data in both research settings as well as open sharing of data through websites such as OpenSNP (Greshake et al. 2014), where users may, under the guise of anonymity (Venkatesaramani et al. 2021), upload their genome as sequenced by companies such as 23andMe, intending for the data to be useful to researchers and other individuals alike. Commonly used measures to protect individual privacy when sharing genomic data in research settings often involve sharing limited information, for example, queries about the presence or absence of particular single-nucleotide variants (SNVs) (Fiume et al. 2019), or summary statistics about single-nucleotide polymorphisms (Sankararaman et al. 2009; MacArthur et al. 2021). Such limited-information releases were initially thought to sufficiently protect individual privacy. However, both presence/absence queries, as well as summary statistics, have been shown to be susceptible to membership inference (MI) attacks using likelihood ratio tests (LRTs) (Homer et al. 2008; Shringarpure and Bustamante 2015). Typically, it is assumed that an attacker has access to a set of target genomes and leverages statistical tests to infer whether each target individual is present in the data set. This information about membership in the database can, in turn, be linked to other sensitive information about the individual, based on the metadata associated with the data release. For instance, a data set may be known to contain individuals with a certain clinical condition (e.g., heart condition).

A host of techniques have been proposed over the years to protect the privacy of released genomic data. Most such methods involve some form of data obfuscation or suppression of a subset of the data, where specific techniques include leveraging the theoretical bounds on the power of inference attacks (Sankararaman et al. 2009), federated access control in the form of presence or absence queries (Fiume et al. 2019) or summary statistics (MacArthur et al. 2021), optimization-based and game-theoretic approaches (Wan et al. 2017a,b; Venkatesaramani et al. 2023), and randomization-based techniques, including differential privacy (DP) (Dwork et al. 2006), randomly masking rare alleles (Raisaro et al. 2017), or simply publishing noisy summary statistics. Another approach based on falsifying responses for rare alleles was proposed in 2018 (Bu et al. 2018). However, this method assumes that allele presence or absence queries are made sequentially, whereas we focus on the case in which all positions are queried at once. These techniques often do not allow the data custodian to trade off utility and privacy at sufficiently high resolution, requiring a large utility loss in order to guarantee a desired level of privacy. Further, such methods usually use only one type of data obfuscation—either adding noise to queries or summary statistics or suppressing data—in order to achieve their privacy goals, whereas there may be significant utility gains from combining these, as we show below.

We consider membership-inference attacks on genomic data sharing in two summary release models: (1) summary statistics, in which a service publishes, for example, alternate allele frequencies (AAFs) for each genomic variant (Sankararaman et al. 2009), and (2) simple “minor allele existence” responses that allow users to query whether a particular allele (e.g., a “*C*” or a “*G*”) is present at a specific position on the genome (e.g., position 1,234,567 on Chromosome 8), as performed by the Beacon services introduced by the Global Alliance for Genomics and Health (GA4GH) in 2015 (Global Alliance for Genomics and Health 2016). We make two contributions. First, we present a novel model of defense through the lens of an optimization problem that combines query suppression and addition of noise to query responses and explicitly trades off utility and privacy. Second, we present two models of attack that inform the privacy component of the utility; the first of these is the conventional approach making use of a fixed threshold to determine membership, whereas the second makes membership claims adaptively to the defense by choosing a threshold that well separates the individuals in the protected service from those in a reference population. Third, we present highly scalable algorithmic approaches for both problem settings, as well as for both threat models, and show that our approaches improve on the state of the art *both* in utility and privacy while easily scaling to problem instances involving more than 1.3 million genomic variants.

## Methods

### Preliminaries

We first introduce the reader to the necessary background regarding how the data are represented mathematically for the sake of problem formulation. This is followed by a summary of the MI attacks investigated in this paper. We then provide a description of linkage disequilibrium, a measure of correlation across alleles, and of how an attacker may exploit such knowledge to infer which alleles are amended by defensive measures. Before elaborating upon our model, we describe the notion of DP, a sophisticated technique to preserve privacy in summary data that one of our methods relies on. Finally, we identify bounds on privacy risk based on the average dissimilarity between genomes.

#### Data representation

A SNV is a position on the genome where the allele present differs across the population. In this study, all SNVs considered are assumed to be biallelic; namely, there are two possible alleles that may be shown for a given SNV. Either one of the possible alleles may be considered a reference allele for a given study, and the other is said to be the alternate allele. The fraction of individuals with the alternate allele in a data set *D* of *n* individuals is referred to as the AAF, which we denote by *p*_*j*_ for the j^th^ SNV. The frequency of the alternate allele in a reference population of individuals not in the data set (we call this $\bar{D}$) is denoted as ${\bar{\;p}}_{j}$. Here, we are only concerned with whether or not an individual has an alternate allele and not with whether both alleles at the chosen position are the alternate allele. Therefore, the binary variable *d*_*ij*_ denotes whether individual *i* has at least one alternate allele (*d*_*ij*_ = 1) at position *j* and *d*_*ij*_ = 0 otherwise. The total number of SNVs in the data set is denoted *m*. Let *Q* refer to a set of *m* SNV positions that can be queried. Let γ be the genomic sequencing error rate (usually on the order of 10^−6^). The probability that no individual in *D* has an alternate allele (equivalently, all individuals have the reference allele) at position *j* is given by $R_{n}^{j}={\left(1-{\bar{\;p}}_{j}\right)}^{2n}$. Let the summary release be represented by the vector *x*. In the case of Beacons, *x* is binary, with *x*_*j*_ = 1 if $\exists \;i\in D,d_{ij}=1$ and *x*_*j*_ = 0 otherwise, indicating the presence or absence of an alternate allele in the data set. In the case of summary statistics, the release is a vector of AAFs; therefore, *x*_*j*_ ∈ [0, 1]. Table 1 summarizes the notation used throughout this paper.

**Table 1.**
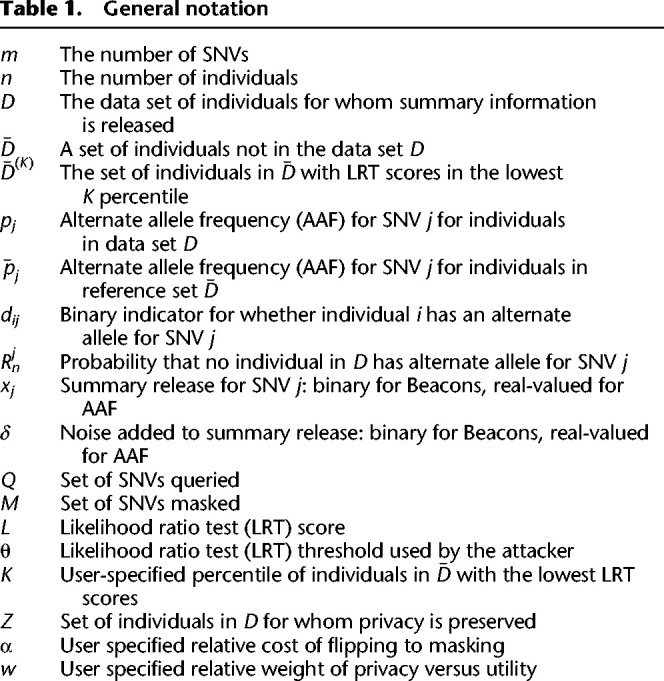
General notation

#### MI attacks

The two MI attacks considered in this work are based on LRT statistics. These statistics represent the relative likelihood of an individual *i* being in the data set *D* upon which the summary release was computed to the likelihood that *i* is in a reference population. An attacker is assumed to have a set of target genomes, for which MI is performed using released summary information, namely, Beacon responses or AAFs. In the case of Beacon responses, we use the LRT statistic proposed by Raisaro et al. (2017), which in turn extends the attack originally proposed by Shringarpure and Bustamante (2015). The original attack assumed AAFs to be drawn from the beta distribution, whereas the extended version uses real AAFs instead. The statistic is computed as follows: Let *T* be the set of target individuals. Then, given the vector *x* of Beacon responses to queries *Q*, the LRT score for individual *i* ∈ *T* is

(1) $$L(Q,d_{i},x)=\underset{\;j\in Q}{\sum }\;d_{ij}\left(x_{j}\log\frac{1-R_{n}^{j}}{1-\gamma R_{n-1}^{j}}+(1-x_{j})\log\frac{R_{n}^{j}}{\gamma R_{n-1}^{j}}\right).$$

The LRT statistic for AAFs is calculated in a similar fashion and was proposed by Sankararaman et al. (2009). Suppose that we have chosen to release AAFs (real-valued vector *x*) for set *Q* of SNVs. An attacker who is in possession of the genome of a particular individual *i* can calculate the log-likelihood ratio statistic for *i* as follows:

(2) $$L(Q,d_{i},x)=\underset{\;j\in Q}{\sum }\;d_{ij}\log\frac{{\bar{\;p}}_{j}}{x_{j}}+(1-d_{ij})\log\frac{1-{\bar{\;p}}_{j}}{1-x_{j}}.$$

#### Linkage disequilibrium

So far, the attack models presented are based on the assumption that SNVs are independent. However, in practice, an adversary may be able to exploit correlations between SNVs to infer Beacon responses for SNVs that are modified. The LD (Von Thenen et al. 2019) is one formal measure of such correlations. Given two loci (a locus is the position of a certain gene on a chromosome) with alleles {*A*, *a*} and {*B*, *b*}, respectively, the LD is computed as *LD* = *P*(*AB*) − *P*(*A*)*P*(*B*). The value of the linkage disequilibrium coefficient (henceforth called LD) lies between −0.25 and 0.25, where larger values are indicative of higher-than-random association of the specified alleles.

We evaluate the effect of an adversary accounting for correlations as follows. In this situation, for each SNV that is either flipped or masked, the adversary identifies SNVs that are correlated with the target SNV. Because computing the LD for all pairs of 1.3 million SNVs is very computationally expensive, we model an adversary who computes correlations with a fixed number of SNVs on either side of the SNV of interest in the data set. We consider two SNVs to be correlated if their LD is above a certain threshold, *t*_*LD*_, limiting our evaluation to highly correlated SNV pairs. Given a flipped or masked SNV *j*, let *N*_*LD*_(*j*) be the set of SNVs that are positively correlated with *j*. If at least 75% of SNVs in *N*_*LD*_(*j*) have a *yes* Beacon response, then the Beacon response for SNV *j* is inferred to be *yes* as well. We show in a subsequent section that only SNVs with *yes* responses are masked/flipped. Having inferred responses for a subset of the flipped/masked SNVs, the adversary now recomputes LRT scores and makes MI claims using the fixed-threshold or adaptive-threshold models as described above. Observe that this evaluation is worst case in the sense that we only perform the above correlation attack for SNVs that *we know* have been flipped or masked, rather than *all* SNVs, as would be performed in an actual attack.

#### DP and the Laplace mechanism

DP is a popular privacy-preserving data-sharing technique based on the principle that the distribution of responses computed on two data sets that only differ in one entry should be similar.

Formally, a randomized algorithm *f* is *ε*-differentially private if for any two data sets *D* and *D*′ differing in one entry (i.e., *D*′ omits one record from *D*) and for all possible subsets *F* of the image of *f*,

(3) $$\frac{P(f(D))\in F}{P(f(D^{\prime }))\in F}\leq \varepsilon .$$

##### Unbounded risk

In practice, the value of *ε* is used to trade off utility and privacy, with a smaller value of *ε* corresponding to a greater degree of privacy at the cost of utility. For aggregate queries such as means over columns, as is the case with AAFs, a simple mechanism to achieve DP is to add random noise sampled from a Laplace distribution to each SNV's AAF. Laplacian noise with a scale of Δg/ε, where Δ*g* is the sensitivity of the function *g* (in our case, *g* is the mean for each SNV), satisfies the requirements for ϵ-DP (Dwork et al. 2006). The sensitivity of a function is defined as $\max{\left\|g(D)-g(D^{\prime })\right\|}_{1}$, where *D* and *D*′ differ in one entry (i.e., *D*′ omits one individual from *D*). For a given SNV in a data set of *n* individuals, *x*_*ij*_ can be either one or zero. Therefore, the maximum possible difference between means over columns differing in one entry is 1/*n*. As the data set has *m* SNVs, the sensitivity, which is the ℓ_1_ norm of the vector of size *n* with each entry being 1/*n*, is $\Delta g=\frac{m}{n}$. Therefore, adding Laplacian noise centered at zero and with a scale of m/nε satisfies ε-DP.

##### Bounded risk

Although the above measure of sensitivity provides theoretical worst-case privacy guarantees, two genomic sequences rarely—if ever—tend to be completely dissimilar in terms of alternate allele composition. The above measure of sensitivity, owing to the large number of SNVs considered (on the order of 1.3 million), forces the user to choose a very large value of ε (on the order of 100,000 or above when no SNVs are masked) to retain sufficient practical utility. Such large values of ε, in turn, offer lower privacy guarantees. Therefore, we also consider a measure of sensitivity in the average-case scenario, in which the numerator consisting of the total number of SNVs, *m*, when calculating sensitivity is replaced by the average number of bits by which a sequence in the data set differs from each sequence in the reference population. We refer to this as bounded sensitivity, and on our data, this measure is an order of magnitude smaller than worst-case sensitivity (on the order of 150,000 when no SNVs are masked).

### Model

#### Defending against MI attacks

In this work, we make a significant departure from existing approaches to preserving the privacy of individuals when sharing genomic summary statistics in two ways. First, we explicitly enable tradeoffs between privacy (in the sense of protection from MI attacks) and utility (in terms of the extent of modification of the summary statistics). Second, we consider two defensive strategies to mitigate the privacy risks presented by MI attacks, namely, suppression (masking) of Beacon responses and the addition of noise to query responses or allele frequencies. In the case of Beacon responses, the addition of noise takes the form of falsifying responses to queries (claiming that a particular alternate allele is not present in the data set when, in fact, it is). This query-flipping approach is standard in much of the prior literature, with various strategies applied to select the subset of SNVs to flip (Tang et al. 2016; Raisaro et al. 2017; Wan et al. 2017a; Cho et al. 2020; Venkatesaramani et al. 2023). In contrast, masking Beacon responses (Sankararaman et al. 2009) is a less explored strategy. This is likely because it has a considerably larger impact on utility. In the case of allele frequencies, we add real-valued noise to the published frequencies, which is a hallmark of methods based on DP concepts, but we differ from prior literature in that ours is the first work that combines the addition of noise with suppression of SNVs. Formally, let *M* be the subset of SNVs that are masked or suppressed. We assume that the data recipients only observe Q∖M, rather than *Q* and *M* separately; consequently, the choice of *M* does not in itself reveal information about the individuals in the data set. Let *δ* denote the noise added to SNVs in Q∖M. In the case of Beacon responses, let *δ* be a vector, with *δ*_*j*_ = −1 indicating that the response for SNV *j* is flipped and *δ* = 0 indicating otherwise. To flip a query *j* is to return a response 1 − *x*_*j*_ for it, whereas masking *j* implies that it cannot be queried at all. In case of AAF releases, let *δ* denote the real-valued noise added to the AAFs *x* for SNVs in Q∖M.

The LRT score for an individual *i* when set *M* of SNVs is masked and noise *δ* is added to the remaining release is $L(Q\setminus M,d_{i},x+\delta )$. Overloading notation, we use *L*_*i*_(*M*, *δ*) to refer to either data release model henceforth, only making a distinction where mathematically necessary. Finally, we can also write the prediction threshold for whether individual *i* is in the data set *D* as a function of the defense, θ(*M*, *δ*). To ensure privacy is preserved for an individual *i* ∈ *D* is to ensure *L*_*i*_(*M*, *δ*) − θ(*M*, *δ*) ≥ 0. Given *M* and *δ*, we define *Z*(*M*, *δ*)⊆ *D* to be the set of individuals for whom masking SNVs in *M* and adding noise *δ* to the rest preserves privacy, namely,

(4) Z(M,δ)={i∈D|L(M,δ)−θ(M,δ)≥0}.

Our goal is to solve the following Summary Stats Privacy Problem (SSPP):

(5) $$\underset{M,\delta }{\min}\;\alpha {\left\|\delta \right\|}_{1}+(1-\alpha )|M|-w|Z(M,\delta )|.$$

where α denotes the relative cost of adding noise compared with masking SNVs, and *w* captures the relative importance of preserving privacy for individuals over the utility of the released summary statistics. Preserving privacy for all individuals often comes at a prohibitive cost to the utility of the released aggregate data and is not always desirable. This approach allows the data custodian to explicitly trade off privacy and utility through a combination of masking and adding noise in a systematic way.

Finally, we consider two threat models, differing in the choice of the prediction threshold, *θ*(*M*, *δ*). The first model involves an attacker who computes the threshold a priori, not accounting for the defense. We also consider an additional *adaptive* attacker model recently introduced by Venkatesaramani et al. (2023) in the context of genomic Beacon services. In this stronger adversarial model, the attacker attempts to identify individuals in the data set, *accounting for the defense* by relying on the separation of LRT scores.

##### Fixed threshold attacks

A naive attacker, who does not account for our attempts to defend against such attacks, chooses a threshold θ, typically to balance false-positive and false-negative rates with respect to some synthetic ground truth data set. A maximum false-positive rate is often used to tune θ (Tang et al. 2016; Wan et al. 2017a). This may be accomplished by simulating Beacons on other publicly available data sets, genomic data that the adversary otherwise has access to, or data synthesized using knowledge of AAFs. The practical implication of this assumption is that we can set the threshold to be a constant, namely, θ(*M*, *δ*) = θ, which does not depend on the subset of SNVs masked or the noise added. As a result, *Z*(*M*, *δ*) = {*i* ∈ *D* | *L*(*M*, *δ*) − θ ≥ 0} for this threat model. This threat model is assumed by most prior work (Sankararaman et al. 2009; Shringarpure and Bustamante 2015; Raisaro et al. 2017). Note that this model does not require the defender to know the threshold θ used by the attacker; a conservative bound will do.

##### Adaptive threshold attacks

On the contrary, the *adaptive* attack model attempts to separate the two populations (individuals in *D* from those who are not) using the separation between their LRT scores *after* the defense has been implemented. Recall that $\bar{D}$ is a set of reference individuals not in the data set *D*. Let ${\bar{D}}^{\left(K\right)}\subset \bar{D}$ be a set of *K* individuals in $\bar{D}$ such that they have the lowest LRT scores. In the *adaptive* attack model, the prediction threshold is calculated as

(6) $$\theta (M,\delta )=\frac{1}{K}\underset{k\in {\bar{D}}^{\left(K\right)}}{\sum }\;L_{k}(M,\delta ).$$

As a result, the set of individuals for whom privacy is protected is $Z(M,\delta )=\{i\in D\;|\;L_{i}(M,\delta )-\frac{1}{K}\sum_{k\in {\bar{D}}^{\left(K\right)}}L_{k}(M,\delta )\geq 0\}$ under the adaptive attacker model. We now present our approach to solving SSPP, under the two threat models discussed above. Note that the noise *δ* that is added to summary statistics is qualitatively different in Beacons compared with AAF summary releases. Recall that although in the former, *δ* is additive noise that codifies whether or not SNVs are flipped; in the latter case of AAFs, *δ* is real-valued. As a result, the two scenarios yield structurally different optimization problems but follow the same general framework as outlined in the Model section. In both cases, we combine the addition of noise with selective suppression of a subset of SNVs.

We begin by rewriting the LRT scores for individual *i* as follows. Let *Q*_1_⊆ *S* be the subset of SNVs for which Beacon response *x*_*j*_ = 1, and let *Q*_0_⊆ *S* be the subset where *x*_*j*_ = 0. Then, the LRT score for individual *i* can be written as

(7) $$L(Q,d_{i},x)=\underset{\;j\in Q_{1}}{\sum }\;d_{ij}A_{j}+\underset{\;j\in Q_{0}}{\sum }\;d_{ij}B_{j}$$

where $A_{j}=\log\frac{1-R_{n}^{j}}{1-\gamma R_{n-1}^{j}}$, and $B_{j}=\log\frac{R_{n}^{j}}{\gamma R_{n}^{j}}$ . In case of AAFs, let $A(x_{j})=\log\frac{{\bar{\;p}}_{j}}{x_{j}},\;\mathrm{and}\;B(x_{j})=\log\frac{1-{\bar{\;p}}_{j}}{1-x_{j}}$. Note that in this scenario, *A* and *B* are functions of *x*_*j*_, instead of constants for each *j* as in the Beacon service. Then the LRT score can be rewritten as

(8) $$L(Q,d_{i},x)=\underset{j}{\sum }\;d_{ij}A(x_{j})+(1-d_{ij})B(x_{j})$$

We note that in the case of Beacons, following the method previously described (Venkatesaramani et al. 2023), we assume that the alternate allele is the minor allele at a given position *j*. This, in turn, allows us to leverage a bound on the genomic sequencing error *γ* to ensure our solution approach never violates privacy previously achieved using an iterative process, as we shortly explain. On the other hand, for AAF releases, we only assume that AAFs are bound by [0.0001, 0.9999] in order to prevent division by zero, and any SNV may be masked in case of AAF summary releases. It was shown by Venkatesaramani et al. (2023) that flipping Beacon responses *x*_*j*_ from zero to one is counterproductive to defending against LRT-based attacks. We now make an analogous observation for masking queries where *x*_*j*_ = 0 for both Beacons and AAF summary statistics.

Proposition 1. *In a Beacon service, for genomic sequencing error, γ* < 0.25*, B*_*j*_ > 0.

Proof. Let ${\bar{\;p}}_{j}$ be the AAF for SNV *j* in the population. Recall that $R_{n}^{j}={\left(1-{\bar{\;p}}_{j}\right)}^{2n}$. As ${\bar{\;p}}_{j}<0.5\;\forall j$, $\frac{R_{n}^{j}}{R_{n-1}^{j}}={\left(1-{\bar{\;p}}_{j}\right)}^{2}\geq 0.25$. Because *γ* < 0.25, $\frac{R_{n}^{j}}{\gamma R_{n-1}^{j}}>1$, and consequently, $B_{j}=\log\frac{R_{n}^{j}}{\gamma R_{n-1}^{j}}>0$.

Proposition 2. *Suppose Beacon response x*_*j*_ = 0 *for SNV j given Beacon data set D*. *Then, masking the SNV can never increase the LRT score for an individual i* ∈ *D, provided* $\gamma <\frac{R_{n}^{j}}{R_{n-1}^{j}}\;\forall j$.

Proof. Consider SNV *j* and an individual *i* ∈ *D*. If *d*_*ij*_ = 0 (i.e., the individual does not have a minor allele at position *j*), masking the SNV makes no difference to the LRT score (contribution of *j* to LRT score is zero when *d*_*ij*_ = 0; for details, see Equation 1). However, when *d*_*ij*_ = 1, suppressing Beacon response *x*_*j*_ changes the contribution of query *j* to the LRT score from $\log\frac{R_{n}^{j}}{\gamma R_{n-1}^{j}}$ to zero. Based on Proposition 1, it can be seen that $\log\frac{R_{n}^{j}}{\gamma R_{n-1}^{j}}>0$. Thus, if SNV *j* is masked the LRT score can only decrease.

#### Masking SNVs

We begin by considering the impact of masking a single SNV on the LRT score. Let *S* be the set of all SNVs, and let *M*⊆ *S* be the subset of SNVs masked. Let $\Delta_{ij}^{M}$ represent the marginal contribution of masking SNV *j* on the LRT score for individual *i*. In case of the Beacon service, masking a SNV *j* changes its LRT score contribution from *d*_*ij*_*A*_*j*_ to zero, as can be observed from Equation 1. Recall that we only mask SNVs where *x*_*j*_ = 1; therefore, if the individual does not have the alternate allele (i.e., *d*_*ij*_ = 0), masking the SNV makes no difference. Therefore, for Beacons, $\Delta_{ij}^{M}=-d_{ij}A_{j}$.

Similarly, in case of an AAF summary release, masking an SNV *j* changes its LRT contribution from *d*_*ij*_*A*(*x*_*j*_) + (1 − *d*_*ij*_)*B*(*x*_*j*_) to zero, as we can observe from Equation 2. Note that in this case, $\Delta_{ij}^{M}$ is also a function of the AAF *x*_*j*_, and therefore, as real-valued noise may be added to SNV *j* as part of our approach before the SNV is masked, $\Delta_{ij}^{M}=-d_{ij}A(x_{j}+\delta_{j})-(1-d_{ij})B(x_{j}+\delta_{j})$. On the contrary, we assume that the subsets of SNVs flipped and masked in the case of Beacons are disjoint.

#### Adding noise to statistics

Next, we consider the addition of noise to the published statistics, for SNVs that are not masked. Let *δ* denote additive noise. In case of the Beacon services, let *δ*_*j*_ = −1 indicate that SNV *j* is flipped; namely, the Beacon response for SNV *j* changes from one to zero. Note that, following the observation by Venkatesaramani et al. (2023), we only flip SNVs where initially *x*_*j*_ = 1. The marginal impact of flipping Beacon response for SNV *j* on the LRT score for individual *i* is $\Delta_{ij}^{F}=d_{ij}(B_{j}-A_{j})$, as we can observe from Equation 1. In case of AAFs, we use the Laplace mechanism defined in the Preliminaries section to add real-valued noise. Thus, in this case, *δ*_*j*_ ∈ [0, 1], and frequencies after the addition of noise are clipped to ensure they are still in the range [0.0001, 0.9999]. In both Beacons and AAFs, the ℓ_1_ norm of *δ* quantifies the total amount of noise added to the summary statistics.

#### Fixed threshold attacks

We begin by presenting our solution for the fixed-threshold attack model, in which privacy is said to be preserved for an individual *i* when their LRT score calculated after suppressing set *M* of SNVs and adding noise *δ* lies above a constant prediction threshold θ, specified exogenously.

Let *z*_*i*_ ∈ {0, 1} be a binary variable corresponding to individual *i*, where *z*_*i*_ = 1 when privacy is preserved for *i* and *z*_*i*_ = 0 otherwise, and define *y*_*j*_ = 1 if SNV *j* is masked (i.e., *j* ∈ *M*) and *y*_*j*_ = 0 otherwise. Then the following optimization problem optimally solves SSPP for the fixed-threshold attacker:

(9) $$\begin{array}{c} \underset{\delta ,y,z}{\min}\;\alpha ||\delta |{|}_{1}+\sum_{j}\left(1-\alpha \right)y_{j}-w\sum_{i}z_{i}\\ \mathrm{subject}\;\mathrm{to}:\\ (L_{i}(M,\delta )-\theta )z_{i}\leq 0\;\forall \;i\in D.\\ y\in {\left\{0,1\right\}}^{m},\;z\in {\left\{0,1\right\}}^{n},\;\delta \in \{\begin{array}{cc} {\left\{-1,0\right\}}^{m},\; & \mathrm{Beacons}\;\\ R^{\left|Q\setminus M\right|},\; & \mathrm{AAFs}\; \end{array} \end{array}$$

In case of Beacons, *δ* is an integer vector, with entries being either −1 or zero, and the above optimization problem assumes the form of an integer linear program (ILP). Although the ILP optimally solves SSPP, it has an exponential worst-case running time with $O(3^{m})$ possible solutions (each SNV in *Q*_1_ can be flipped, masked, or reported truthfully), which poses significant scalability challenges with larger populations of more than millions of SNVs (for an empirical runtime comparison between optimally solving the ILP and our proposed approach, see Supplemental Fig. 13). In case of AAFs, *δ* is real-valued, and thus, the above problem becomes a mixed-integer program (MIP). Much like the ILP, the MIP has difficulty scaling to large problem instances with more than a million SNVs. To address these limitations, we now introduce heuristic algorithms that approximately solve SSPP for Beacons and AAFs.

##### Heuristic–Beacons

We now introduce a simple greedy algorithm to compute approximate solutions to SSPP for Beacon services. The driving idea behind our greedy heuristic is as follows: At each iteration, we choose a SNV for which flipping or masking achieves the highest average marginal contribution per unit cost (of flipping or masking the SNV) to the LRT scores for individuals in the Beacon. For each individual, let *P*_*i*_ be the set of SNVs for which the Beacon response *x*_*j*_ = 1 and the individual's genome has the associated minor allele; namely, *d*_*ij*_ = 1. For *j* ∈ *P*_*i*_, $\Delta_{ij}^{F}$ and $\Delta_{ij}^{M}$ are independent of *i*. Let $\Delta_{j}^{F}=(B_{j}-A_{j})$ and $\Delta_{j}^{M}=-A_{j}$. Then, $\Delta_{ij}^{F}=d_{ij}\Delta_{j}^{F}$, and $\Delta_{ij}^{M}=d_{ij}\Delta_{j}^{M}$. For a chosen query *j* and a subset of individuals *P*⊆ *B*, let *T*_*j*_ = {*i* ∈ *P*|*j* ∈ *P*_*i*_}, which is the set of individuals for which *j* ∈ *P*_*i*_. The average marginal contribution of flipping the query response to SNV *j* per unit cost is

(10) $${\bar{\Delta }}_{j}^{F}(P)=\frac{|T_{j}|\Delta_{j}^{F}}{\alpha |P|}.$$

Similarly, the average marginal contribution of masking a SNV *j* is

(11) $${\bar{\Delta }}_{j}^{M}(P)=\frac{|T_{j}|\Delta_{j}^{M}}{\left(1-\alpha )|P\right|}.$$

At each iteration, we calculate both ${\bar{\Delta }}_{j}^{F}(P)$ and ${\bar{\Delta }}_{j}^{M}(P)$ for every SNV *j*, and either flip or mask the SNV with the highest overall contribution, depending on whether flipping or masking led to it scoring the highest. The number of individuals for whom we thereby guarantee privacy is nondecreasing through each iteration of this algorithm, because flipping or masking SNVs for which *x*_*j*_ = 1 can only increase LRT scores (see Proposition 2). Each time privacy is assured for at least one additional individual, we compare this privacy–utility point to the current best solution (as measured by the objective function in Equation 9), and update it if it improves the objective. We also update *P* to be the set of individuals for whom privacy is not yet assured. The algorithm iterates until privacy is protected for all individuals in the Beacon or until we cannot flip or mask any more SNVs, at which point we return the overall best solution. This idea is formalized in Algorithm 1, which we call Soft-Privacy-Greedy-Binary (SPG-B). For runtime analysis, refer to Supplemental Methods, Section B.

Algorithm 1. **Soft-Privacy-Greedy-Real-Binary (SPG-B)**

**Input:** A set of individuals *i* ∈ D, subset *P*_*i*_ and LRT score η_i_ for each individual, marginal contributions of flipping/masking $\Delta_{j}^{F}$ and $\Delta_{j}^{M}$ for each SNV, a set of queries *Q* and threshold θ, weight parameter *w*, relative cost of flipping α.

**Output:** Subset of queries *F*⊆*S* to flip, subset of queries *M*⊆*S* to mask.

**Initialization:** *F* = Ø, *M* = Ø, *C* = Ø, *F*_*t*_ = Ø, *M*_*t*_ = Ø, *U* = ∞

**while** (D\C) = Ø **do**

 Set *l* = 0, *N* = −1, *d* = −1.

** for** *j* ∈ (*Q*\ (*F* ∪ *M*)) **do**

  Set *T*_*j*_ = {*i* ∈ (*D*\*C*)| *j* ∈ *P*_*i*_} Set ${\bar{\Delta }}_{j}^{F}=\Delta_{j}^{F}\frac{\left|T_{j}\right|}{\alpha |D\setminus C|}$

  Set ${\bar{\Delta }}_{j}^{M}=\Delta_{j}^{M}\frac{\left|T_{j}\right|}{\left(1-\alpha )|D\setminus C\right|}$

  **if** ${\bar{\Delta }}_{j}^{F}>N$ **then**

   Set $N={\bar{\Delta }}_{j}^{F}$

   Set *l* = *j*

   Set *d* = 0

**  if** ${\bar{\Delta }}_{j}^{M}>N$ **then**

   Set $N={\bar{\Delta }}_{j}^{M}$

   Set *l* = *j*

   Set *d* = 1

 **if** *d* == 0 **then**

  Set *F*_*t*_ = *F*_*t*_ ∪ *l*

 **if** *d* == 1 **then**

  Set *M*_*t*_ = *M*_*t*_ ∪ *l*

 **for** *i* ∈ (*D*\*C*) **do**

  **if** $\sum_{\;j\in F}\Delta_{ij}^{F}+\sum_{\;j\in M}\Delta_{ij}^{M}+\eta_{i}\geq \theta$ **then**

   Set *C* = *C* ∪ *i*

 *U*_t_ = α|*F*_*t*_| + (1−α)|*M*_*t*_|−*w*|*C*|

 **if** *U*_*t*_ ≤ *U* **then**

 Set *U* = *U*_*t*_

 Set *F* = *F*_*t*_

 Set *M* = *M*_*t*_

**return** *F*,*M*

##### Heuristic–AAFs

We now introduce an alternating optimization algorithm that approximately solves SSPP, combining masking of AAFs for a subset of SNVs with adding Laplacian noise to the rest.

The outline of the algorithm is as follows. At each step, we alternate between adding noise to SNVs that have not yet been masked, such that it minimizes the objective in Equation 9, and masking SNVs in order of their average marginal contribution to the population's LR scores, in an attempt to increase utility. The average marginal contribution of masking a SNV *j* is simply the mean over the marginal contributions for all individuals; namely, ${\bar{\Delta }}_{j}^{M}=\frac{1}{n}\sum_{i\in D}\Delta_{ij}^{M}.$

Because in the fixed-threshold model, we require that each individual's score lie *above* a specified threshold, we aim to increase LRT scores. Thus, we rank SNVs to mask in order of their average marginal contributions. For α ≫ (1 − α), masking is preferred over adding noise, in essence, sharing a smaller subset of cleaner data as opposed to sharing all SNVs with high obfuscation. Masking SNVs should necessarily continue to minimize the objective function until privacy is violated for an individual previously covered as a result of encountering large positive values of Δ_*j*_. However, continuing to mask in this manner is suboptimal because it does not allow us to explore possible intermediate solutions; for example, masking fewer SNVs and adding slightly higher noise may provide a better privacy–utility tradeoff in many cases.

Our algorithm proceeds as follows. We first add noise to all SNVs by sampling from the Laplace distribution such that it best optimizes the objective in Equation 9. This may be performed in one of two ways: (1) computing the objective over a preselected set of values of *ε* or (2) performing a binary search over possible values of *ε*, assuming convergence when the difference between two considered values of *ε* in the search is sufficiently small. Although the latter is more systematic, it is also slower and performs poorly (see Supplemental Methods, Section C; Supplemental Figs. 1, 2), whereas the former approach is fully parallelizable and produces good results, as long as the choices for the set of candidate *ε* values are reasonable. We therefore use the first approach in the rest of this work.

Having added Laplacian noise, we then mask a set of *t* SNVs in the order of their average marginal contribution to LR scores, calculated after adding noise. The value of *t* that we use is chosen to balance computation time and the near-optimality of the solution. Specifically, a smaller value of *t* implies a larger number of candidate solutions explored but with a runtime inversely proportional to *t*.

At the end of this cycle, we repeat the noise-addition and masking processes in an alternating fashion, each time adding noise to the SNVs that remain unmasked with the scale of the Laplacian distribution accordingly adjusted. Algorithm 2, which we call the Soft-Privacy-Greedy-Real (SPG-R) approach, provides full details about the implementation of our method. In the Supplemental Methods, Section C, we present an alternate implementation of the SPG-R algorithm that leverages problem structure to reduce redundant computations and that uses parallel processing in order to significantly reduce runtime.

Algorithm 2. **Soft-Privacy-Greedy-Real (SPG-R)**

**Input:** A set of individuals *i* ∈ *D*, marginal contributions of masking ${\bar{\Delta }}_{j}^{M}$ for each SNV, a prediction threshold θ, weight parameter w, relative cost of adding noise α, number of SNVs to mask per iteration *t*, set *E* of candidate DP parameters, AAFs *x* for individuals in *D*, and $\bar{\;p}$ for individuals in reference set $\bar{D}$.

**Output:** Subset of SNVs *M* ⊆ *S* to mask, real-valued noise vector *δ*.

**Initialization:** *M* = Ø, *C* = Ø, *U* = ∞, *δ* = 0, *c* = 0, $\Delta^{S}=$ Sort $\left({\bar{\Delta }}_{j}^{M}\right)$, *M*_*t*_ = Ø

**Function** GetLR(*d*_*i*_, *δ*, *M*):

** return** $\Sigma_{\;j\in S\setminus M}d_{ij}\log\frac{{\bar{\;p}}_{j}}{x_{j}+\delta_{j}}+(1-d_{ij})\log\frac{1-{\bar{\;p}}_{j}}{1-(x_{j}+\delta )}$

**while** *S*\*M*_*t*_ ≠ Ø **do**

 Set *U*_*t*_ = ∞, *δ*_t_ = 0, *C*_*t*_ = Ø

** for** *ε* ∈ *E* **do**

  Set *δ*_*ε*_ = Laplacian(0, $\frac{\left|Q\setminus M_{t}\right|}{n\varepsilon }$)

  Set *C*_*ε*_ = Ø

  **for** *i* ∈ *D* **do**

   **if** GetLR(*d*_*i*_, *δ*_*ε*_, *M*_*t*_) ≤ θ **then**

    Set *C*_*ε*_ = *C*_*ε*_∪*i*

  Set *U*_*ε*_ = *α*||*δ*_*ε*_||_1_ + (1−*α*)|*M*_*t*_|−*w*|*C*_*ε*_|

  **if** *U*_*ε*_ ≤ *U*_*t*_ **then**

   Set *U*_*t*_ = *U*_*ε*_

   Set *δ*_t_ = *δ*_*ε*_

   Set *C*_*t*_ = *C*_*ε*_

** if** *U*_*t*_ ≤ *U* **then**

  Set *U* = *U*_*t*_

  Set *δ* = *δ*_t_

  Set *M* = *M*_*t*_

 Set *c*_*t*_ = 1

** while** *ct* ≤ *t* **do**

  Set $M_{t}=M_{t}\cup \Delta_{c}^{s}$

  Set *c* = *c* + 1

  Set *c*_*t*_ = *ct* +1

 Set *U*_*t*_ = *α*||*δ*_t_||_1_ + (1−*α*)|*M*_*t*_|−*w*|*C*_*t*_|

** if** *U*_*t*_ ≤ *U* **then**

  Set *U* = *U*_*t*_

  Set *δ* = *δ*_t_

  Set *M* = *M*_*t*_

**return** *M*, *δ*

#### Adaptive threshold attacks

In the *adaptive* threshold scenario, the goal is to ensure that the LRT scores of individuals in *D* and $\bar{D}$ (i.e., those not in the data set) remain sufficiently well-mixed. Recall from the Preliminaries section that the prediction threshold in this setting is $\theta (M,\delta )=\frac{1}{K}\sum_{k\in {\bar{D}}^{\left(K\right)}}L_{i}(M,\delta )$, which is the average LRT score for a set of *K* individuals in $\bar{D}$ with the lowest LRT scores. Then similar to Equation 9, we can formulate this as an optimization problem in the context of adaptive attacks.

(12) $$\begin{array}{c} \underset{\delta ,y\in {\left\{0,1\right\}}^{m},z\in {\left\{0,1\right\}}^{n}}{\min}\;\alpha ||\delta |{|}_{1}+\sum_{j}\left(1-\alpha \right)y_{j}-w\sum_{i}z_{i}\\ \mathrm{subject}\;\mathrm{to}:\\ (L_{i}(M,\delta )-\theta (M,\delta ))z_{i}\leq 0\;\forall \;i\in D.\\ \delta \in {\left\{-1,0\right\}}^{m}\left(\mathrm{Beacons}\right);\;\delta \in R^{m}\left(\mathrm{AAFs}\right) \end{array}$$

This structure allows us to extend our algorithms used for the fixed-threshold scenario, with one change: Instead of sorting SNVs by $\Delta_{j}^{M}$ or $\Delta_{j}^{F}$, we now sort the SNVs by $\Delta_{j}^{M(K)}=\Delta_{j}^{M}-\frac{1}{K}\sum_{k\in {\bar{D}}^{\left(K\right)}}\Delta_{kj}^{M}$ and $\Delta_{j}^{F(K)}=\Delta_{j}^{F}-\frac{1}{K}\sum_{k\in {\bar{D}}^{\left(K\right)}}\Delta_{kj}^{F}$, respectively. In the adaptive threshold model, with Beacons, $\Delta_{j}^{M(K)}$ and $\Delta_{j}^{F(K)}$ may be negative and may be detrimental to privacy achieved in prior iterations of our greedy algorithms. As such, masking and flipping are, respectively, restricted to those SNVs in which these quantities are strictly positive.

#### Linkage disequilibrium

To defend against an attacker who leverages correlations to infer flipped/masked SNVs in a Beacon, we introduce a direct extension to our proposed Soft-Privacy-Greedy approach. Specifically, whenever an SNV *j* with known correlations is flipped or masked, all SNVs correlated to it (the set *N*_*LD*_(*j*)) are also flipped or masked, respectively. To capture the corresponding utility loss while deciding which SNV to flip or mask, we modify the Soft-Privacy-Greedy algorithm as follows. For each SNV *j*, we amend the marginal contribution of flipping *j* to ${\bar{\Delta }}_{j}^{F}(P)=\frac{T_{j}\Delta_{j}^{F}}{\alpha |P||N_{LD}(j)|}$, as well as the marginal contribution of masking SNV *j* to be ${\bar{\Delta }}_{j}^{M}(P)=\frac{T_{j}\Delta_{j}^{M}}{\left(1-\alpha )|P||N_{LD}(j)\right|}$. The algorithm then proceeds as before, with the added condition that, any time a SNV *j* is picked such that $N_{LD}(j)\neq \emptyset$, all SNVs in *N*_*LD*_(*j*) are also correspondingly flipped or masked. We refer to this modified algorithm as Soft-Privacy-Greedy-LD ( SPG-LD).

## Results

### Experimental design

#### Data set and metrics

Our experiments were conducted on a data set of 1,338,843 SNVs on Chromosome 10, made available by the 2016 iDASH workshop on privacy and security (Tang et al. 2016). The data consist of genomes of 400 individuals for whom summary statistics are to be released (i.e., the set *D*), and 400 individuals who are not part of this group (the set $\bar{D}$). This data set was derived from the 1000 Genomes Project (The 1000 Genomes Project Consortium 2015) and is sufficiently large to show the scalability of our approach, while using only Chromosome 10 makes it practical to work with in terms of memory footprint. All experiments were conducted on a PC with an AMD Threadripper 3960X CPU and 128-GB RAM, running Ubuntu 22.04. We measure utility as $100[1-(\alpha {\left\|\delta \right\|}_{1}+(1-\alpha )|M|)/m]\text{\%}$, where *α* is the relative cost of adding noise to masking SNVs, *M* is the set of SNVs masked, and *m* is the number of SNVs. We define privacy as the percentage of individuals for whom privacy is preserved under each respective attacker model, as is the standard assumption in prior literature. The same measure of privacy was thus applied to all baselines when tuning for best utility for the sake of a fair comparison.

#### Runtime analysis

Both SPG-B and SPG-R have a worst-case time complexity of $O(m^{2}n)$ (for details, see Supplemental Methods, Section B). In practice, as the number of SNVs outpaces the number of individuals by several orders of magnitude, the impact of increasing the number of individuals is negligible. In the case of SPG-R, solutions that minimize the objective function are computed for a range of candidate values of *ε*. Although the number of candidate values for *ε* is small compared with the number of SNVs and individuals, the serial execution of operations for each candidate *ε* adds significant runtime overhead in practice, which can be greatly reduced by leveraging parallel processing, in combination with heuristics derived from problem structure. This provided motivation for the implementation of a parallel version of SPG-R, which is used for all experiments presented in this paper. The full details of the parallel implementation are provided in the Supplemental Methods, Section C.

#### Baselines–SPG-B

We compare our approach (SPG-B) to four state-of-the-art baselines. The first is strategic flipping (SF), in which SNVs are flipped in decreasing order of their differential discriminative power, as proposed by Wan et al. (2017a), followed by a local search. We also compare to a modified version of SF, which we call SFM, in which the adaptive threshold definition of privacy is used when applicable. The second is random flipping (RF) (Raisaro et al. 2017), in which unique alleles in the data set (i.e., only one individual has the allele) are randomly flipped by sampling from a Binomial distribution. The third is a differentially private (DP) mechanism proposed by (Cho et al. 2020), which offers plausible deniability for each Beacon response. Fourth, we compare to the marginal-impact greedy (MIG) approach by Venkatesaramani et al. (2023), noting that this approach guarantees privacy for all individuals. For each baseline, we consider a variation that selects SNVs as described by the method but masks the SNVs instead of flipping them. None of the baselines allow us to combine flipping and masking into a single strategy.

#### Baselines–SPG-R

We compare the performance of the proposed SPG-R algorithm with three baselines: (1) only adding noise using the Laplace mechanism (standard DP), (2) *masking* only, and (3) the *linkage* approach proposed by (Sankararaman et al. 2009), which greedily selects a subset of SNVs in linkage equilibrium in order of utility up to the maximum allowed power of the LR test. We note that we use the parallel version of SPG-R as detailed in the Supplemental Methods, Section C, throughout this work.

### Fixed threshold attacks

#### SPG-B

We begin by considering fixed-threshold attacks on Beacons, with the prediction threshold θ = −250. For SPG-B, we measure performance in the utility–privacy space by varying the relative weight of privacy to utility *w* ∈ [0.01, 10]. For DP and RF, we vary their respective parameters (noise parameter *ε* and probability *p*, respectively). Note that when approaches rely solely on masking SNVs, the only solution for threshold θ above a certain positive value is to shut the Beacon down (responses that are initially zero are not masked) as the maximum LRT score attainable for an individual *i* is $\theta \geq \sum_{\;j\in Q_{1}}d_{ij}B_{j}$, where *Q*_1_ is the subset of SNVs for which Beacon response is one, and *d*_*ij*_*B*_*j*_ is the marginal contribution of flipping the response for SNV *j* for individual *i*. Therefore, here we show results for a negative value of θ for which a solution is guaranteed. Results for θ = −750 as well as special cases in which all approaches including SPG-B are restricted to either flipping or masking SNVs are similar and provided in the Supplemental Methods, Section D (refer to Supplemental Figs. 3–5).

**Figure 1.**
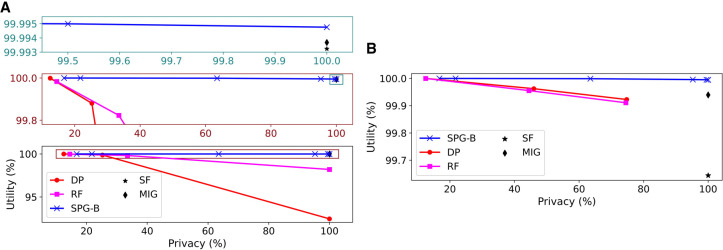
Utility–privacy plots for the fixed threshold attack model for Beacons, compared with baselines. (*A*) θ = −250, baselines only flip SNVs. Zoomed in portions shown in *top* two subplots. (*B*) θ = −250, baselines only mask SNVs.

Figure 1 compares the performance of SPG-B to the baseline approaches when (1) the baselines only flip SNVs and (2) the baselines only mask SNVs, respectively, as none of the baselines allow us to use a combination of the two. The key observation is that the proposed approach Pareto dominates the baselines, as the ability to both flip and mask SNVs provides an additional level of flexibility. In Figure 1A, the improvement over both DP and RF is particularly substantial in terms of utility. MIG and SF offer slightly lower utility than our approach when the privacy of all individuals is protected, but they do not permit solutions that can explicitly trade off utility for privacy. Figure 1B tells a similar story, in which it can be seen that the difference in utility between SPG-B and MIG increases from ∼0.001% (when MIG flipped SNVs) to ∼0.05% (in the masking case in which θ = −250). This difference in utility corresponds to tens of thousands of more SNVs masked by MIG than SPG-B (as there are more than 1.3 million SNVs in the data set and α ≫ (1 − α)).

#### SPG-R

In a similar fashion, we compare the parallel variant of SPG-R to the three baselines in the fixed threshold setting. We set the prediction threshold θ = 0 in these experiments, as this was found to be the threshold that best separated the two populations before the defense was applied. In contrast to Beacons, we explore a wider range of values of α, the relative cost of masking to adding noise, as the ℓ_1_ norm of real-valued noise *δ* is orders of magnitude smaller on average than it is in Beacons. We vary the relative weight of privacy to utility *w* in the range [0.1, 10000]. For DP, linkage, and masking, we choose the privacy–utility point that best optimizes the SSPP objective function for each value of *w*, ensuring fair comparisons. All results involving random noise are averaged over five runs. Here, we only present results for unbounded risk; those for bounded risk are similar and provided in the Supplemental Methods, Section F (refer to Supplemental Figs. 8, 9). Additional results about the impact of increasing population sizes on SPG and DP are presented in Supplemental Figures 11 and 12.

We select the DP noise parameter ϵ for the Laplace mechanism from {10,000, 50,000, 100,000, 500,000, 1 million, 5 million, 10 million}. Although at first it seems like these values are very large compared with the parameters used in practice (on the order of one to 10), this is explained by the fact that the data sets used in practice have nowhere near the number of variables we are dealing with. With more than 1.3 million SNVs, using a smaller value of *ε* would induce a prohibitive amount of noise (for details on how noise scales with the number of SNVs *m* and *ε*, see Preliminaries Section) that would essentially void the system of any utility.

**Figure 2.**
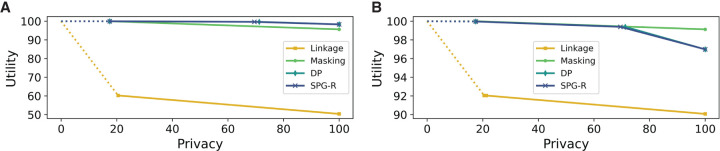
Utility–privacy plots for the fixed threshold attack model for AAF releases, compared with baselines. (*A*) θ = 0, α = 0.5. (*B*) θ = 0, α = 0.9.

Figure 2 considers the scenario in which the relative cost of adding noise to masking α = 0.5 and α = 0.9. When α = 0.5, SPG-R outperforms the linkage and masking methods and has comparable performance to DP (Fig. 2A). When α = 0.9, on the other hand, masking outperforms other methods (because it has far lower cost than adding noise), with both SPG-R and DP having comparable performance to masking, and one another (Fig. 2B).

### Adaptive threshold attacks

Next, we consider the adaptive threshold attacker, with *K* = 10 (here, this refers to the *K* lowest percentile in terms of LRT scores). Yet again, we present results by varying the relative weight of privacy-to-utility *w* as before, and the respective parameters for the considered baselines.

#### SPG-B

**Figure 3.**
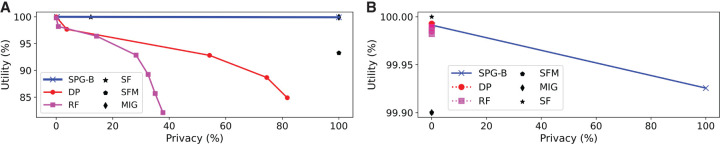
Utility–privacy plots for the adaptive threshold attack model for Beacons, compared with baselines. (*A*) *K* = 10, baselines only flip SNVs. (*B*) *K* = 10, baselines only mask SNVs.

Figure 3 presents results in this setting, in which we observe that again SPG-B Pareto dominates the baselines and is comparable to MIG, but now by a much larger margin than in the fixed threshold setting. In Figure 3A, although SF has better utility than the remaining baselines, it offers very low privacy. If we restrict the baselines to masking only, Figure 3B shows that SPG-B once again outperforms all baselines. The reason is evident from the plot itself: A masking-only strategy is insufficient to guarantee privacy against an adaptive-threshold attacker. Results for *K* = 5, as well as settings in which all approaches including SPG-B are restricted to flipping or masking, are similar and provided in the Supplemental Methods, Section E (refer to Supplemental Figs. 6, 7).

#### SPG-R

**Figure 4.**
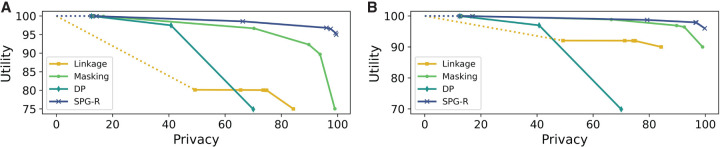
Utility–privacy plots for the adaptive threshold attack model for AAF releases, compared with baselines. (*A*) *K* = 10, α = 0.75. (*B*) *K* = 10, α = 0.9.

Figure 4 presents results for *K* = 10, for α = 0.75 and α = 0.9. In contrast to the fixed threshold setting, here, SPG-R dominates all baselines. When α = 0.9 (Fig. 4A), namely, adding noise is relatively expensive, masking produces similar performance when *K* = 5 (see Supplemental Methods, Section E); however, once *K* is increased to 10 (Fig. 4B), the problem can no longer be solved using masking alone, and SPG-R dominates it by a significant margin. DP and linkage offer much lower utility on average compared with only masking SNVs or using SPG-R.

### Linkage disequilibrium

**Figure 5.**
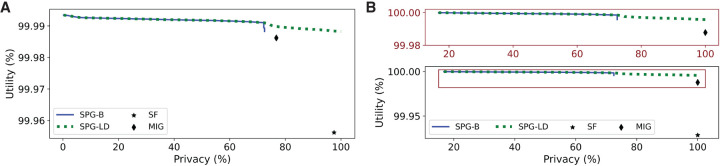
Fixed threshold attack model, in which the attacker leverages correlation data, and baselines only flip SNVs. (*A*) θ = 1000, baselines only flip SNVs. (*B*) θ = −250, baselines only mask SNVs.

Finally, we consider attacks on Beacons that leverage correlations between SNVs. We assume that a pair of SNVs is correlated if their LD-coefficient is above 0.2. LD is measured within a span of 250 SNVs on either side of each target SNV. The attack was found to have no impact on DP and RF, so these are omitted from the following plots. Figure 5 presents results in the fixed-threshold setting. Figure 5A shows that the attack has a small impact on the privacy of MIG and a significant impact on SF and SPG-B, limiting SF to 77% − 80%, and SPG-B to around 75% when baselines flip SNVs for prediction threshold θ = 1000. When baselines mask SNVs (Fig. 5B), the correlation attack has no impact on SF and MIG but reduces the maximum privacy achieved by SPG-B to ∼73% when θ = −250. In both cases, SPG-LD successfully defends against the correlation attack, achieving 100% privacy for large values of *w* while dominating the baselines.

**Figure 6.**
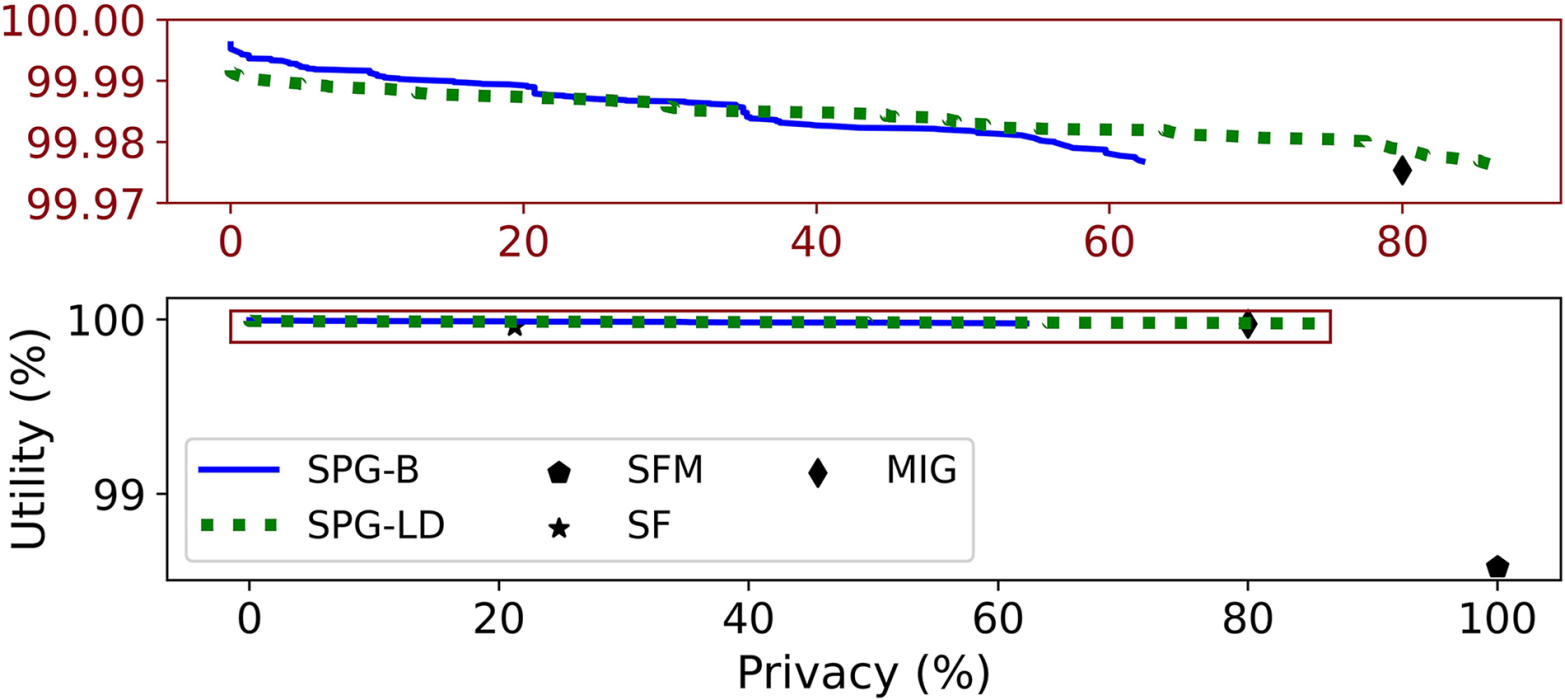
Adaptive threshold attack model, in which the attacker leverages correlation data, baselines flip SNVs.*K* = 5, baselines only flip SNVs.

Figure 6 presents results in the adaptive threshold case, when *K* = 5 and baselines flip SNVs. As before, the attack affects SPG-B, SF, and MIG, although to a greater extent in this setting compared with the fixed threshold model. The privacy achieved by SF drops to ∼22% and that of MIG drops to ∼80%. The privacy achieved by SFM is unaffected by the attack; however, SFM yields much lower utility than our proposed methods. The privacy achieved by SPG-B is reduced to ∼63% when the attacker uses correlations. The modified approach, SPG-LD, dominates all approaches in terms of utility. In addition, it raises the privacy to ∼88%. However, it fails to achieve privacy for all individuals, even with very large values of *w*. None of the baselines preserve privacy for any individuals solely by masking SNVs. Therefore, the performance of SPG-LD versus SPG-B is the same as in Figure 6, such that we do not present new results for the adaptive threshold setting. For additional experiments with relaxed constraints on the choice of the subset of SNVs masked *M*, see the Supplemental Methods, Section G (see Supplemental Fig. 10).

In the case of AAFs, adding Laplacian noise provides privacy protections under the assumption that SNVs are independent, although this may not be the case in practice. However, as indicated above, our results indicate that DP mechanisms are fairly robust to attacks that leverage correlation in the data in the Beacon case. Additionally, although correlations in the Beacon case may enable an attacker to infer the true Beacon response, in the case of AAFs, only noisy statistics are exposed for the set of SNVs that are not masked. Thus, we expect SPG-R to also be robust to attacks leveraging correlation information.

## Discussion

In this study, we presented a formalization to the problem of finding the optimal privacy–utility tradeoff when defending against membership-inference attacks on genomic summary releases (Beacon services and summary statistics), allowing—unlike prior studies—for the defense to combine masking of SNVs and the addition of noise to best balance the two while accounting for the relative cost of adding noise compared with suppressing responses. In the case of Beacons, we further evaluate an extension of the proposed approach against a more powerful attacker model in which correlations between SNVs are exploited to infer modified responses. We present a simple, yet principled greedy algorithm for both release models to discover the best privacy–utility balance that outperforms prior art, evaluating it against powerful attacks from recent literature. In most cases, our approach outperforms existing methods in terms of privacy, particularly in the context of the adaptive threshold attacker, where our approach protects privacy for nearly all individuals. Our approach preserves privacy in a manner that allows the data to retain the highest utility. Several thousand fewer SNVs are modified by our method in the case of Beacons. In the case of AAFs, masking a small number of SNVs allows us to add noise that is orders of magnitude smaller than traditional DP-based methods to achieve a comparable level of privacy. It should be recognized that our approach does have certain limitations in that it is specific to the MI attacks that leverage an LRT score. More powerful attacks may be devised that defeat our approach.

### Software availability

All of the code and data used in this study are publicly available as the Supplemental Material, as well as at Zenodo (https://doi.org/10.5281/zenodo.7510802).

## Supplementary Material

Supplement 1
