# Supplementary material for: Enabling tradeoffs in privacy and utility in genomic data Beacons and summary statistics
Source: Genome Res. 2023 Jul;33(7):1113–23. doi: 10.1101/gr.277674.123 (PMC10538482; doi:10.1101/gr.277674.123)
Supplement: Supplement 1 [file Supplemental_Methods.pdf]

Supplemental Methods

This supplement provides a comprehensive overview of related work, additional results for the various threat models described in the paper, runtime analysis for the SPG-B algorithm. and a parallel implementation of SPG-R which leverages problem structure to speed up computation.

A Related Work

The prior literature on data privacy spans a host of techniques. There has been a substantial amount of research into mechanisms based on differential privacy (Dwork et al. 2006). Recently, for instance, local differential privacy (LDP) (Erlingsson et al. 2014) has gained popularity, where data is first perturbed locally before an aggregator computes overall database statistics. Wang and colleagues (Wang et al. 2019) proposed novel LDP mechanisms, and extended them to aggregate computations on locally perturbed multidimensional data. Gu et al. (Gu et al. 2020) addressed the potential differences in privacy requirements at each data collection source for LDP with an input-discriminative extension to LDP. Takagi et al. (Takagi et al. 2021) proposed P3GM, a differentially-private generative model based on variational autoencoders to overcome the issue of a large amount of noise injected into high-dimensional data by traditional DP techniques. Xie and colleagues (Xie et al. 2021) extended an encryption scheme used for IP addresses to more general datatypes, combined with a multi-view outsourcing method which generates one utility-preserving view of the data for analysis among several fake indistinguishable views in order to protect privacy.

Attacks specific to Beacon services have evolved since their introduction in 2015. Shringarpure and Bustamante (Shringarpure and Bustamante 2015) illustrated how to leverage likelihood-ratio test (LRT) scores to make membership inference claims from Beacon responses. They specifically used Beacons from the 1000 Genomes Project (1000 Genomes Project Consortium 2015) and the Personal Genome Project (Chervova et al. 2019) and demonstrated that only a small number of queries are sufficient to predict membership. The study assumed that allele frequencies are drawn from a Beta distribution. Raisaro and colleagues (Raisaro et al. 2017) extended the attack using real allele frequencies instead of assuming that allele frequencies are drawn from the Beta distribution. The authors proposed three defense strategies against such a membership inference attack: a) a Beacon alteration strategy, where the Beacon flips all responses for unique alleles (i.e., only one individual in the dataset contains a minor allele at a given position), b) a random flipping strategy, where the Beacon responses for unique alleles are flipped, but randomly by sampling from a binomial distribution, and c) a query-budget strategy, where the contribution of each individual in the dataset to the Beacon responses is used to decide whether the individual's genome will be included in providing a Beacon response to authenticated Beacon users. In the query-budget approach, however, the authors assumed that the genomic sequencing error is 0, in order to simplify analysis. Greedy approaches in this special case with no sequencing error

are discussed in (Venkatesaramani et al. 2021b).

Von Thenen and colleagues (Von Thenen et al. 2019) introduced an allele-inference technique by leveraging linkage disequilibrium between alleles, where a higher-order Markov chain is used to infer alleles at positions of interest from a few correlated SNVs. The study showed that, by inferring hidden SNVs using the proposed technique, an attacker can make membership-inference claims, despite making far fewer queries to the Beacon. This allows the attacker to potentially bypass a query-budget defense, as well as defenses that mask SNVs with smaller minor allele frequencies. A related attack, introduced by Ayoz and colleagues (Ayoz et al. 2021), considers genome reconstruction for evolving Beacons, where Beacon-responses are supplemented with phenotype metadata, and the attacker already knows about an individual's membership. Bu and colleagues (Bu et al. 2021) introduced a haplotype-based membership inference attack that reconstructs haplotypes using allele frequencies, as opposed to relying on a target genome. Samani and colleagues (Samani et al. 2015) introduced am method that relied on high-order SNV correlations to carry out an inference attack on Beacons using Markov models.

At the same time, several defenses against such attacks have been proposed. Wan and colleagues (Wan et al. 2017a) selected a subset of SNVs to flip by defining a differential discriminative power that captures a SNV's marginal contribution to the LRT score. The approach selected SNVs in decreasing order of the proposed metric, followed by a greedy local search to improve utility. Cho and colleagues (Cho et al. 2020) used an approach based on differential privacy to flip Beacon responses, a method that forms one of the baselines we use in this paper. This study relied upon a differentially private geometric mechanism, treating SNVs in an independent manner. Finally, (Venkatesaramani et al. 2021b) proposed greedy algorithms to select a subset of SNVs based on marginal impact, by drawing parallels to the set-cover problem. This study identified several special cases, such as very low DNA sequencing error, and when allele frequencies were assumed to be drawn from the Beta distribution.

B SPG Runtime Analysis

We show that the running time of the proposed SPG-B and SPG-R algorithms is quadratic in the number of SNV queries m and linear in the number of individuals m in the Beacon.

Theorem 3. *The worst-case running time of the* SOFT-PRIVACY-GREEDY-BINARY *algorithm is* $\mathcal{O}(m^2 n)$.

Proof. All operations inside the first *for* loop are constant time, and the loop executes m times, where m is the number of SNVs - therefore yielding a complexity of $\mathcal{O}(m)$. The *if* condition inside the second *for* loop involves a sum over m SNVs, and is therefore an $\mathcal{O}(m)$ operation, and this *for* loop executes at most n times in the worst case, with a total time complexity of $\mathcal{O}(mn)$. The remaining statements within the *while* loop are constant time operations. The outer *while* loop executes at most m times, as all SNVs are flipped or masked in the worst case, and therefore the overall time complexity of SPG-B is $\mathcal{O}(m(m+mn+1)) = \mathcal{O}(m^2 n)$. □

Theorem 4. *The worst-case running time of the* SOFT-PRIVACY-GREEDY-REAL *algorithm is* $\mathcal{O}(m^2n)$.

Proof. The `GetLR` module is linear in the number of SNVs, i.e. $\mathcal{O}(m)$. Statements inside the first *for* loop execute for each candidate value of ε, and the number of candidate values, i.e., $|E|$ is a constant (k). Statements inside the inner *for* loop execute once for each individual, i.e. exactly n times. All `Set` commands are constant time. Thus the inner *for* loop has time complexity $\mathcal{O}(kmn) = \mathcal{O}(mn)$. The while loop executes t times, where t is the number of SNVs to mask per iteration, and has time complexity $\mathcal{O}(t)$. The outer *while* loop iterates over all SNVs in batches of size t, which is essentially linear in m. The overall time complexity of SPG-R, therefore, is $\mathcal{O}(m(mn+t)) = \mathcal{O}(m^2n+mt) \approx \mathcal{O}(m^2n)$, given that $m \gg t$. □

Remark. *In practice, $m \gg n$, therefore we can treat n as a small constant, and the runtime is approximately $\mathcal{O}(m^2)$. We further note that the worst-case running time complexity of MIG and SPG-B is of the same magnitude, as they proceed almost identically, except that in SPG-B we consider marginal impacts of masking, as well as flipping each SNV, and compare the two - which are constant time operations inside a loop that iterates over all SNVs.*

C Improving Runtimes for SPG-R

When optimizing the real valued noise using the Laplace mechanism, we can utilize some structural observations to avoid redundant computations over large matrices which, in turn, can cut down runtimes by orders of magnitude. Our first observation is that the scale of the Laplacian distribution from which random noise is drawn is directly proportional to the number of SNVs that are not yet masked. Recall that the scale of the Laplacian when no SNVs are masked is $m/n\varepsilon$. If a kth fraction of SNVs remains unmasked, the scale of the Laplacian, accordingly, is $m/kn\varepsilon$. Consider the noise added per SNV for a given value of ε, when no SNVs are masked. This same amount of noise is achieved *per SNV* when a kth fraction of SNVs is unmasked, with noise added corresponding to ε/k, and herein lies our first runtime improvement. When a noise sample is drawn from a Laplacian distribution with scale s for a given value of ε, the same noise can be used at scale s/k for a corresponding DP parameter ε/k. While drawing a noise sample is not an expensive operation in itself, this observation allows us to re-use previously computed values for Δ^M_{ij} and $\Delta^{M(K)}_{ij}$ at different (scaled) values of ε as more SNVs are masked. As both Δ^M and $\Delta^{M(K)}$ contain one entry per individual per SNV, re-computing values for a fresh noise sample each time contributes significantly to overall runtime. As long as the choices of ε at the beginning of the algorithm are well-spread out (over multiple orders of magnitude, as the best solutions may involve masking a significant fraction of SNVs), we can avoid re-computing Δ^M and $\Delta^{M(K)}$ as we change the scale of the Laplacian, instead assuming the noise to be generated for a correspondingly scaled value of ε. We note that the ℓ_1 norm of the noise for the objective function would still have to be recomputed over only the SNVs that remain unmasked, but this is a relatively inexpensive operation.

Algorithm 3: The SPG-R (parallel) Algorithm

Input: A set of individuals $i \in D$, a prediction threshold θ, weight parameter w, marginal contributions of masking Δ^M, relative cost of adding noise α, number of SNVs to mask per iteration t, set E of candidate DP parameters, AAFs x for individuals in D and $\bar{p}$ for individuals in reference set $\bar{D}$.

Output: Subset of SNVs $M \subseteq Q$ to mask, real-valued noise vector δ.

Initialization: $M = \emptyset$, $C = \emptyset$, $U = \infty$, $\delta = 0$

Function `GetLR`(x_i, Δ, M)**:**

  return $\sum_{j \in Q \setminus M} -\Delta_{ij}$

for $\varepsilon \in E$, **in parallel do**

  Set $\delta^{\varepsilon} = \text{Laplacian}(0, \frac{|Q|}{n\varepsilon})$, $M_{\varepsilon} = \emptyset$, $c_{\varepsilon} = 0$

  $\Delta^{\varepsilon}_{ij} = -d_{ij} \log \frac{\bar{p}_j}{x_j + \delta^{\varepsilon}_j} - (1 - d_{ij}) \log \frac{1 - \bar{p}_j}{1 - (x_j + \delta^{\varepsilon}_j)}$

  Set $\Delta^{\varepsilon}_j = \frac{1}{|D|} \sum_{i \in D} \Delta^{\varepsilon}_{ij}$

  Set $\Delta^{S\varepsilon} = \text{SORT}(\Delta^{\varepsilon}_j)$

  while $Q \setminus M_{\varepsilon} \neq \emptyset$ **do**

    Set $C_{\varepsilon} = \emptyset$

    for $i \in D$ **do**

      if GETLR$(x_i, \Delta^{\varepsilon}, M_{\varepsilon}) \leq \theta$ **then**

        Set $C_{\varepsilon} = C_{\varepsilon} \cup i$

    Set $U_{\varepsilon} = \alpha ||\delta_{\varepsilon}||_1 + (1 - \alpha)|M_{\varepsilon}| - w|C_{\varepsilon}|$

    if $U_{\varepsilon} \leq U$ **then**

      ACQUIRELOCK(U, δ, M)

      Set $U = U_{\varepsilon}$

      Set $\delta = \delta^{\varepsilon}$

      Set $M = M_{\varepsilon}$

      RELEASELOCK(U, δ, M)

    Set $ct = 1$

    while $ct \leq t$ **do**

      Set $M_{\varepsilon} = M_{\varepsilon} \cup \Delta^{S\varepsilon}_c$

      Set $c = c + 1$

      Set $ct = ct + 1$

return M, δ

The second structural observation about our solution approach is that the privacy-utility points explored for a given set of candidate ε values are independent of w, the relative importance of guaranteeing privacy over preserving utility. In Algorithm 2, threads for parallel processing are initialized after masking every t SNVs. Even if we use global variables (one instance of noise δ and Δ^M_{ij} or $\Delta^{M(K)}_{ij}$, depending on the attacker model, for each value of ε), the repeated creation and synchronization of threads before masking the next set of SNVs can add significant overhead. To deal with this, we re-formulate our search on a per-ε basis, where each thread masks SNVs locally. Threads still share access to the matrix x, but avoid repeated function calls and synchronization wait times. Moreover, we can save all candidate solutions explored by recording $||\delta||, \sum_j y_j$, and $\sum_i z_i$ under either attack model, and for any value of α and w, and find the best solution in linear time over the saved candidate solutions, which are in turn linear in the number of SNVs. The algorithm, SPG-R (parallel), that takes advantage of these improvements, is provided in Algorithm 3.

OPTIMIZING OVER ε

We compare the two variants of SPG-R (binary and parallel) under the harder of the two attacker models - the adaptive

threshold setting. Supplemental Figure 1 shows the relative performance of the two variants when $K = 5$ and $K = 10$.

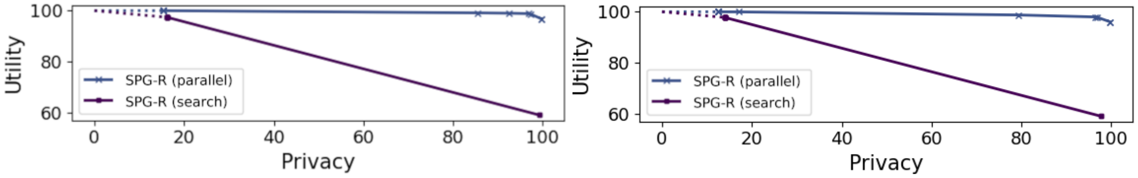

(a) K=5 (b) K=10

Supplemental Figure 1: Relative performance of SPG-R (binary) and SPG-R (parallel) under the adaptive threshold model

Binary search was initialized with $\varepsilon \in \{10K, 10M\}$, and candidate values for SPG-R (parallel) were selected from $\varepsilon \in \{10K, 50K, 100K, 500K, 1M, 5M, 10M\}$. We can observe that SPG-R (parallel) significantly outperforms SPG-R (binary) in utility-privacy tradeoff, likely because the latter does not explore useful tradeoff points.

Empirical Runtime Comparison Next, we compare the runtimes for the various methods used in Supplemental

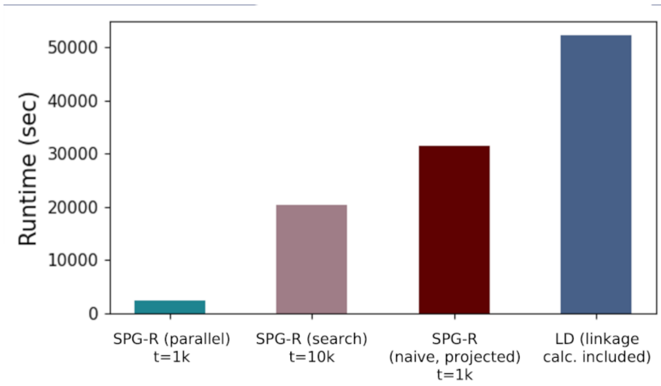

Supplemental Figure 2: Runtime comparison between SPG-R variants and baselines.

Figure 2. The number of SNVs masked in one iteration (t) used for each approach is indicated in the plot. We also compare the runtimes to a projected estimate of a non-parallel naive implementation of SPG-R, where solutions over the various candidates for ε are sequentially computed. Runtime for DP is omitted because it is too small compared to the rest. The runtime for SPG-R (binary) is an order of magnitude larger than SPG-R (parallel), even when masking 10 times the number of SNVs in each iteration ($t = 10K$), taking about 5.5 hours in practice. SPG-R (binary) with $t = 1000$ can therefore be expected to take in excess of 55 hours. The estimated runtime for naive implementation of SPG-R is calculated by multiplying the average runtime of SPG-R (parallel) with the number of threads and adding some marginal overhead for thread creation and synchronization. Runtime for Linkage includes the time taken to compute linkage disequilibrium coefficients for pairs of SNVs using a sliding window of 500 SNVs (250 on either side of each SNV), which takes about 15 hours in practice, although we note that this is a parallelizable problem with

scope for shared data structures, and the computation only needs to be done once.

D Additional Results - Fixed Threshold

First, we present additional results where SPG-B combines flipping and masking, while baselines solely flip or mask SNVs for $\theta = -750$ in Fig 3. Performance, in this case, is similar to the results for $\theta = -250$, in that SPG-B Pareto dominates all baselines, and shows significant improvements in utility over DP and RF - both when baselines only flip SNVs (Supplemental Figure 3a) or mask SNVs (Supplemental Figure 3b).

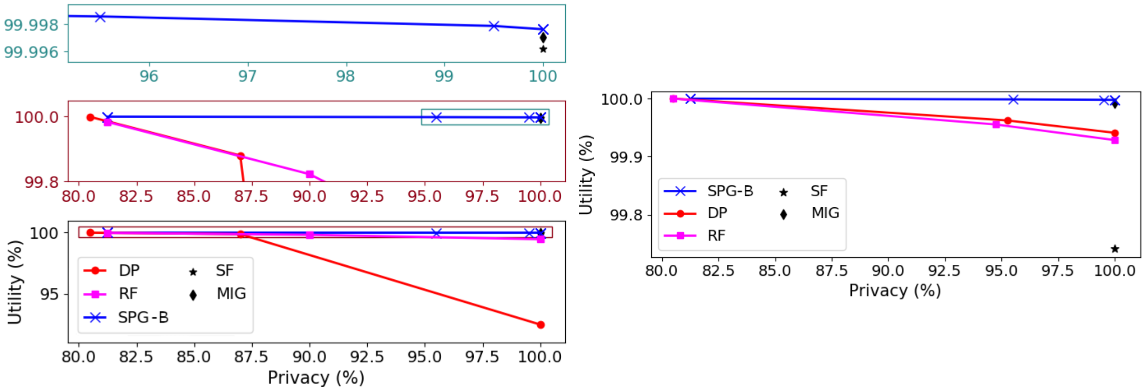

(a) $\theta = -750$, baselines only flip SNVs

(b) $\theta = -750$, baselines only mask SNVs

Supplemental Figure 3: Utility-privacy plots for the fixed threshold attack model for Beacons, compared to baselines.

Special Case: Only Flipping SNVs

We now consider the special case where all approaches, including SPG-B, are restricted to flipping SNVs. This represents a scenario where suppressing Beacon responses may be impractical. In this setting, in addition to SPG-B and the various baseline methods shown in the more general setting, we also present the optimal solution computed using CPLEX (IBM ILOG Cplex 2009), an ILP-solving toolkit. Since the original ILP in (12) is unable to scale to a search space consisting of 1.3 million SNVs, we restrict the ILP to search for an optimal utility-privacy balance over the SNVs identified by MIG in this setting (on the order of 10^2). In this scenario, the higher the value of θ, the more SNVs the defender has to flip to guarantee privacy. A higher value of θ thus more clearly demonstrates the differences in utility across the methods, and therefore we present results for $\theta = 0$ and $\theta = 1000$.

Supplemental Figure 4 shows results in this setting when $\theta = 0$ and $\theta = 1000$. SPG-B again Pareto dominates DP, RF, and SF. SF guarantees privacy for all individuals while offering much lower utility. MIG guarantees privacy for all individuals with a slightly lower utility when $\theta = 1000$, dropping further when $\theta = 0$. In practice, the difference between the performance of MIG and SPG-B arises from flipping about 10 additional SNVs to guarantee privacy for only a single individual in the dataset. For a very large value of the weight parameter w, SPG-B produces the same

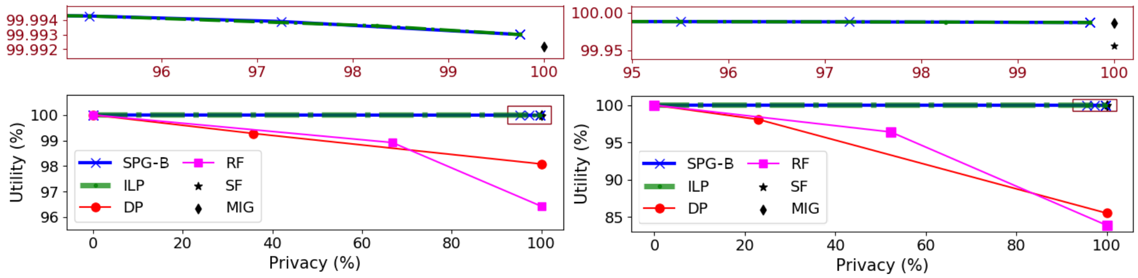

(a) $\theta = 0$ (b) $\theta = 1000$

Supplemental Figure 4: Fixed threshold attack model when all approaches only flip SNVs.

solution as MIG in this setting.

Special Case: Only Masking SNVs We now consider the alternative case where SNVs are only masked. This setting also serves to demonstrate the greater loss of utility that must be tolerated to achieve privacy for all using just masking. Note that the impact of masking a Beacon response is smaller than that of flipping it.

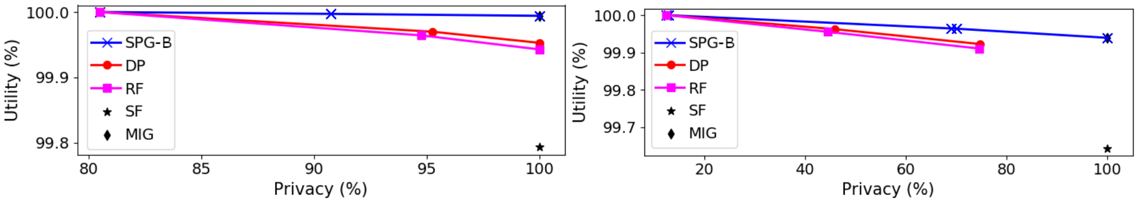

(a) $\theta = -750$ (b) $\theta = -250$

Supplemental Figure 5: Fixed threshold attack model when all approaches only mask SNVs.

Supplemental Figure 5 presents the results when the θ prediction threshold is set to -250 and -750. Yet again, we observe that SPG-B Pareto dominates all baselines, with MIG offering comparable utility when privacy of all individuals is necessarily guaranteed. Comparing the performance of SPG-B between Supplemental Figure 5 and Supplemental Figure 1 for $\theta = -250$, it can be seen that choosing to flip a small number of SNVs and masking the remaining greatly improves utility.

E Additional Results - Adaptive Threshold

Here, we present some additional results in the adaptive threshold setting for both SPG-B and SPG-R. First, we compare the performance of SPG-B to various baselines, when the threshold is set to the mean of $K = 5$ lowest percentile of LRT scores, where baselines either flip or mask SNVs, while SPG-B combines both. From Supplemental Figure 6, we can observe that SPG-B once again dominates all baselines. None of the baselines offer any privacy when they are restricted to masking SNVs in this case.

Special Case: Only Flipping SNVs

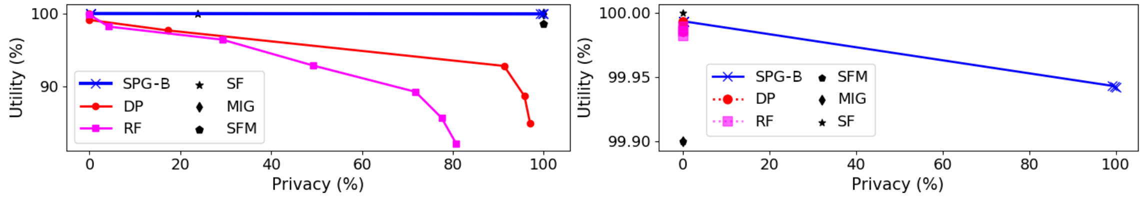

(a) $K = 5$, baselines only flip SNVs

(b) $K = 5$, baselines only mask SNVs

Supplemental Figure 6: Utility-privacy plots for the adaptive threshold attack model for AAF releases, compared to baselines.

Finally, we present results in the case where all methods, including SPG-B, only flip SNVs. Fig.7 compares SPG-B to the baselines. It can be seen that SPG-B offers a better privacy-utility balance than all methods, except for MIG when privacy of all individuals is to be guaranteed. In comparison to Figs. 3 and 6, it can be seen that SPG-B has a slightly lower utility. This further illustrates the value of applying a method that uses both flipping and masking.

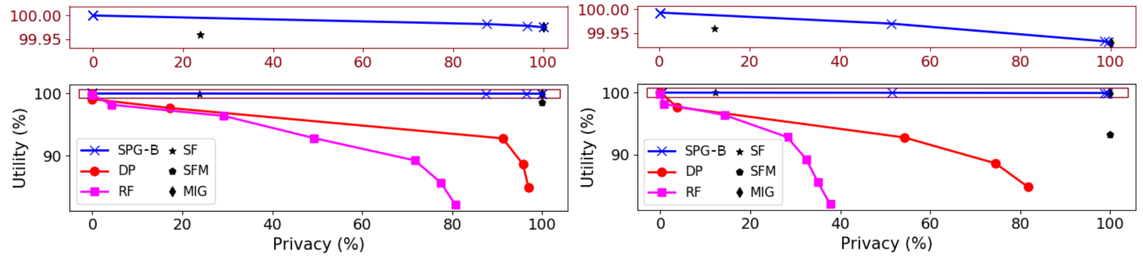

(a) $K = 5$

(b) $K = 10$

Supplemental Figure 7: Adaptive threshold attack model, where all approaches only flip SNVs.

F Additional Results - Bounded Risk

Here, we compare the performance of SPG-R (parallel) to the various baselines, under the assumption of bounded risk, where the sensitivity of the mean query is calculated in the average case instead of the worst case, and correspondingly the scale of the Laplacian depends on the average number of bits by which a genome in the dataset differs from those not in the dataset. On our data, the average sensitivity is 148515, which is an order of magnitude smaller than the number of SNVs.

This has no qualitative impact on the best solutions found by our approach, except that a correspondingly smaller value of DP parameter ε is now used to generate the same amount of noise. Because our candidate ε values were well spread out ($\varepsilon \in \{10K, 50K, 100K, 500K, 1M, 5M, 10M\}$), our approach works well without any modifications.

Fixed Threshold Attacks

Supplemental Figure 8 compares SPG-R to the various baselines under the fixed threshold attack model, when $\theta = 0$ for $\alpha = 0.5$ and $\alpha = 0.9$. As is the case with unbounded risk, SPG-R dominates all baselines when $\alpha = 0.5$, but as

masking gets relatively cheaper compared to adding noise, SPG-R is dominated by a pure-masking strategy, with the difference being more pronounced in the bounded risk scenario.

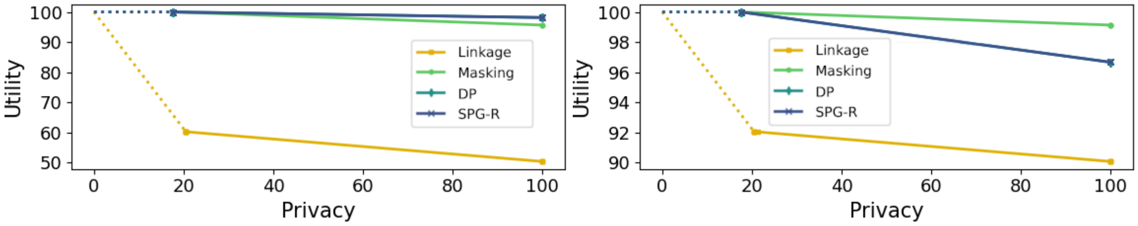

(a) $\alpha = 0.5$ (b) $\alpha = 0.9$

Supplemental Figure 8: Performance of SPG-R compared to baselines in the fixed threshold setting (bounded risk) $\theta = 0$.

Adaptive Threshold Attacks

The performance with bounded risk in the adaptive threshold setting yet again qualitatively mirrors the results in the unbounded risk setting, with SPG-R dominating all baselines, as we can observe from Supplemental Figure 9.

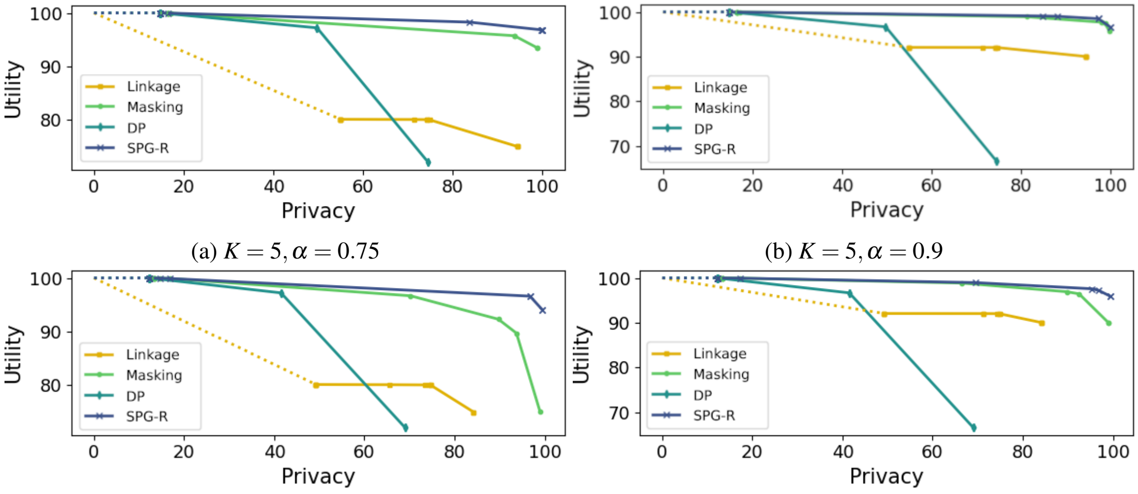

(a) $K = 5, \alpha = 0.75$ (b) $K = 5, \alpha = 0.9$

(c) $K = 10, \alpha = 0.75$ (d) $K = 10, \alpha = 0.9$

Supplemental Figure 9: Performance of SPG-R compared to baselines in the adaptive threshold setting (bounded risk) for $K = 5$ and $K = 10$.

G Additional Results - LD

In the adaptive threshold case, recall that flipping or masking is restricted to SNVs where $\Delta_{ij}^{F(K)} \geq 0$ and $\Delta_{ij}^{M(K)} \geq 0$ respectively for all individuals in the Beacon. When a SNV j is flipped or masked by *SPG-LD*, there may be SNVs in $N_{LD}(j)$ for which these inequalities may not hold true, and are therefore not flipped or masked. These SNVs however may still be used to infer flipped or masked Beacon responses by measuring correlations.

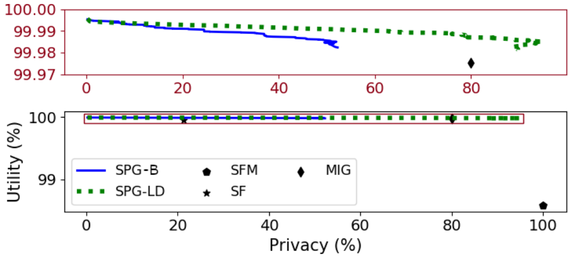

Supplemental Figure 10: Adaptive threshold attack model, where the attacker leverages correlation data, and baselines only flip SNVs. The Greedy approach permitted to flip or mask all SNVs. $K = 5$

The correlation attack had no impact on SPG-BB when $K = 10$. None of the SNVs picked by SPG-B in this setting to either flip or mask had correlations with other SNVs within a sliding window of 500 SNVs (250 on each side). Therefore, we ran a second set of experiments that neglects these inequality constraints, essentially allowing Beacon responses for all SNVs to be flipped or masked, with the consequence that privacy achieved in early iterations of the greedy algorithms may be reduced by later flips or masks. Supplemental Figure 10 presents the results. While the performance of SF, SFM and MIG are unchanged compared to the previous setting in Supplemental Figure 6, notice that for both SPG-B and SPG-LD, the privacy starts decreasing after a point, as more and more SNVs are flipped or masked. The maximum privacy achieved by the modified greedy algorithm accounting for correlations, SPG-LD, is around 90%. This is a marginal increase compared to the previous setting.

H Impact of Increasing Population Sizes

Here, we present additional results comparing the relative performance of DP to SPG-B and SPG-R for Beacons and summary statistics as we increase the number of individuals in the dataset (n). With the available hardware, we were able to experiment with summary statistics for datasets consisting of up to 600 individuals. Supplemental Figure 11 shows that neither approach is significantly affected by population size in the case of Beacons. Supplemental Figure 12 suggests that, while neither approach is affected in the fixed-threshold setting for summary statistics, the relative performance of DP deteriorates in terms of utility as the number of individuals increases in the adaptive threshold case. The slight difference in performance trends between these figures and the results presented in the main paper is due to randomly splitting the universe of all individuals ($D \cup \bar{D}$) into two sets of varying sizes here, as compared to working with a particular 400-400 split in previous settings.

I Empirical Runtime - ILP vs SPG-B

Finally, we present empirical results comparing the running times of the ILP and SPG-B (refer Section D for details). Because the ILP was only feasible over a small set of SNVs chosen by MIG, we restrict SPG-B to the same set of SNVs to ensure a fair comparison. For both approaches, running time was only measured for the steps involved in

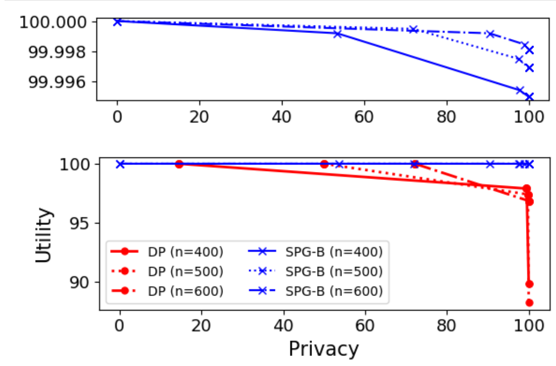

(a) $\theta = -250$, DP flips SNVs

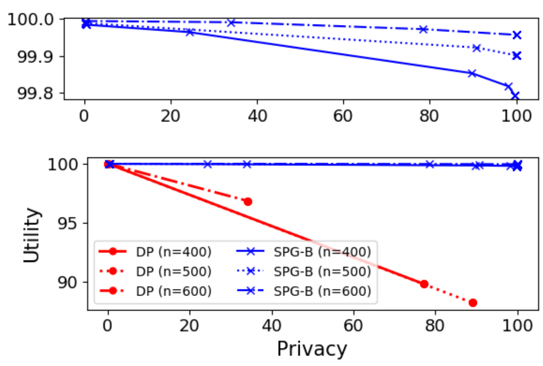

(b) $K = 10$, DP flips SNVs

Supplemental Figure 11: Relative performance of DP and SPG-B with increasing dataset size (n). Zoomed-in portions shown on top.

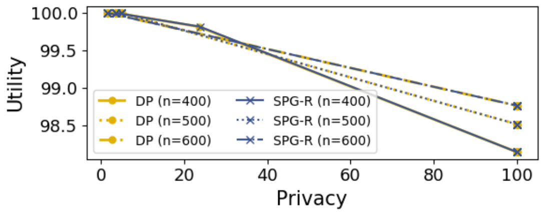

(a) $\theta = 0, \alpha = 0.5$

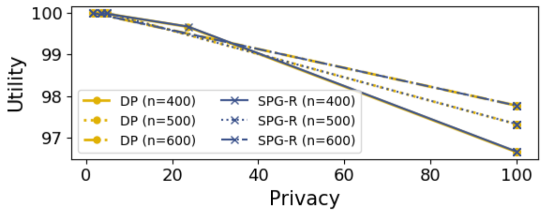

(b) $\theta = 0, \alpha = 0.9$

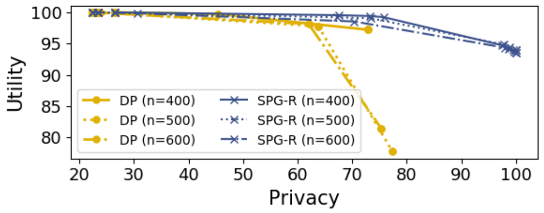

(c) $K = 10, \alpha = 0.75$

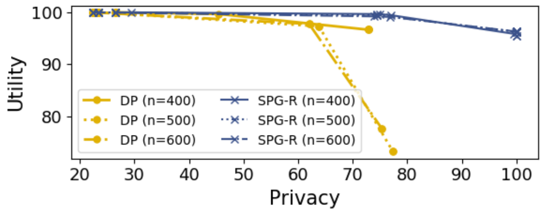

(d) $K = 10, \alpha = 0.9$

Supplemental Figure 12: Relative performance of DP and SPG-R with increasing dataset size (n)

solving the optimization, ignoring all initialization and pre-computation steps. This analysis was performed on a 2018 MacBook Pro with an Intel i7 processor and 16 GB of RAM.

Supplemental Figure 13 depicts that the running time for the ILP is between 2 to 6 orders of magnitudes larger, depending on the weight parameter. For small weights, the optimal solution is to do nothing, and for large enough weights, the optimal solution is to guarantee privacy for everyone - these are solutions that a branch-and-bound approach is expected to arrive quickly at - which is what IBM CPLEX uses in order to compute optimal solutions. For weight parameters between these two extremes, we expect that the branching would need to continue to a greater depth, leading to the spike in running time at $w = 0.2$. On the other hand, SPG-B continues flipping or masking SNVs until all individuals are covered, regardless of w, the weight is only used to update the current best solution which is eventually returned. Therefore the running time is constant across all weights.

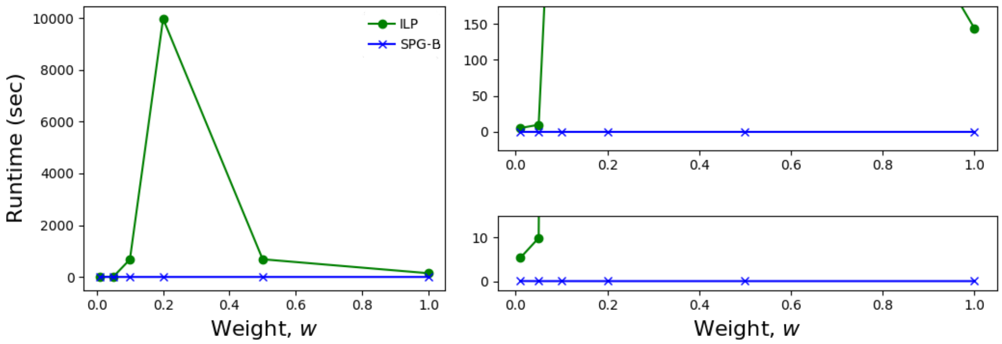

Supplemental Figure 13: Empirical Comparison of running times for ILP and SPG-B (using only SNVs identified by MIG)
